# Estimating the critical shear stress for incipient particle motion of a cohesive soil slope

**DOI:** 10.1038/s41598-022-13307-w

**Published:** 2022-06-13

**Authors:** Xingyu Yuan, Fei Ye, Wenxi Fu, Lina Wen

**Affiliations:** 1 State Key Laboratory of Hydraulic and Mountain River Engineering, College of Water Resource and Hydropower, Sichuan University, Chengdu, 610065 People’s Republic of China; 2 Sichuan Highway Planning, Survey, Design and Research Institute Ltd, Chengdu, 610041 People’s Republic of China

**Keywords:** Environmental sciences, Natural hazards

## Abstract

The critical shear stress is a vital reference indicator for soil erosion. Soil erosion will occur when soil slope suffers from a exceed shear stress, and then causing soil loss and destruction of soil structure. In this work, an equation was proposed based on the force equilibrium of a single particle to estimate the critical shear stress for incipient particle motion of a cohesive soil slope. This formula is characterized by its physical significance, and the critical shear stress for incipient slope soil motion can be easily calculated when the soil properties and the slope angle are known. Moreover, the seepage-runoff coupled model and the excess shear stress equation are introduced in this paper. Two parameters, namely the weight of rushed soil particles and the discharge of water, must be measured in the scouring tests. Through linear regression, the tested *τ*_c_-values were obtained to validate the *τ*_c_-values calculated by the formula derived from the critical shear stress. In addition, two other formulas were compared with the derived formulas, which considered more parameters with physical significance. Finally, the influence of all parameters on the critical shear stress was analyzed: the porosity of the soil, the specific gravity of the soil and the slope gradient had less influence on the critical shear stress; the critical shear stress was negatively influenced by the particle diameter and positively influenced by the internal friction angle of the soil.

## Introduction

Soil erosion is a global environmental problem that seriously threatens the stability of slopes and reduces the fertility of soils. At present, rainfall has been recognized as the main triggering factor for soil erosion, as it generates runoff on the slope surface and carries away soil particles. In the field of soil erosion, the erosion rate, which introduces the excess shear stress equation (see Eq. 1), is an important evaluation indicator^1,2^.

1 $$\varepsilon = k_{{\text{d}}} \left( {\tau_{{\text{a}}} - \tau_{{\text{c}}} } \right)^{a} ,$$

where *ε* is the erosion rate by weight (kg/(m^2^·s)), *k*_d_ is the erodibility coefficient (s/m), *τ*_a_ is the surface shear stress acting on the soil boundary (Pa), *τ*_c_ is the critical shear stress (Pa), and the exponent *a* (dimensionless) is often taken to be 1.0.

The erosion rate *ε* was found to be linearly correlated with the surface shear stress *τ*_a_ in Eq. (1) and in-situ experiments were required to determine the *k*_d_- and *τ*_c_-values^3–5^. Several diverse empirical equations for estimating *τ*_c_ have been derived, which are related to the plasticity index, the mean particle diameter, and the percentage of clay by weight^6^. Furthermore, the relevant parameters, such as plants^7^, rainfall intensity^8^ and slope gradient^9^, have also been studied.

Shields (1936) first proposed a dimensionless shear stress criterion to describe the incipient sediment motion^10^. In recent decades, incipient sediment motion has rapidly developed into a branch of river dynamics with the criterion of shear stress^11,12^ or shear velocity^13,14^. Regardless of which criterion is adopted to describe incipient sediment motion, some formulas were derived through force analysis for a single particle^13,15^, while others were concluded through a series of experiments^16,17^. These achievements have made great progress when the soil layer is approximately horizontal, but they cannot be directly applied to slope soil particles. In fact, slope runoff is driven by gravity, and the runoff depth is closely connected to the shear velocity^18^. Based on the force analysis of slope soil particles, the formula for incipient sediment motion of shallow soil slopes can be obtained.

On the other hand, studies on incipient sediment motion still pose a severe problem in that the criteria for judging incipient sediment motion vary from person to person. Four different bed shear conditions for a sedimentary bed were characterized with no transport, weak transport, medium transport, and general transport, where the shear stress with general transport was defined as the critical shear stress^19^. Wilcock and Southard considered the critical shear stress to be approximately equal to the shear stress that produces a small transportation rate^20^. Choi and Kwak analyzed three different incipient modes of particles: rolling, sliding, and lifting^15^. Shvidchenko et al. took the fractional transport rate as an indicator for judging the incipient conditions^21^. Singh et al. used visual observations along with the quantitative measurement of transport rates as an incipient criterion^16^. The incipient standards described above are unquantifiable and diverse, which results in measured critical shear stresses that may not turn out to reflect the actual conditions of the critical shear stress.

Direct measurement of the critical shear stress requires complex experimental equipment. Normally, the critical shear stress can be indirectly measured through scouring tests by using the excess shear stress equation. The results are empirical and lack of physical significance. The main contents in this paper include: (1) proposing a theoretical formula to estimate the critical shear stress of soil slope, which is based on the soil properties (i.e., soil unit weight, porosity, particle size, specific gravity, and internal friction angle) and the slope angle; (2) introducing the seepage-runoff coupling model and the excess shear stress equation to validate the derived formula of the critical shear stress; (3) conducting the deep analysis to explain how are the soil properties and the slope angle influence the critical shear stress.

## Methods

### Theory

#### Expression derivation of the critical shear stress

**Figure 1:**
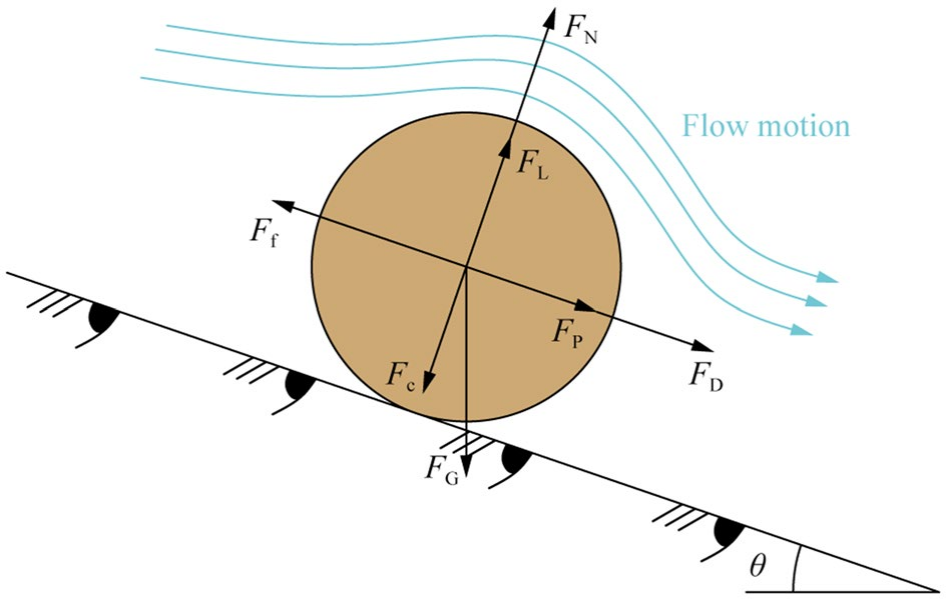
Force analysis for a single particle on the surface of a soil slope.

In this section, we derive a new formula for the critical shear stress based on the force equilibrium of a single particle (Fig. 1).

In addition to gravity, the normal force between particles and cohesion, the particle is also subjected to forces induced by water flow, including buoyant force, seepage force, drag force and uplift force (Fig. 1).

The expressions for effective gravity *F*_G_, uplift force *F*_L_ and drag force *F*_D_ are given as follows^12,22^:

2 $$F_{{\text{G}}} = \frac{{\uppi }}{6}\left( {\gamma^{\prime}_{{\text{s}}} - \gamma_{{\text{w}}} } \right)d^{3} ,$$

3 $$F_{{\text{L}}} = \frac{{\uppi }}{8}C_{{\text{L}}} \rho_{{\text{w}}} d^{2} u_{{\text{b}}}^{2} ,$$

4 $$F_{{\text{D}}} = \frac{{\uppi }}{8}C_{{\text{D}}} \rho_{{\text{w}}} d^{2} u_{{\text{b}}}^{2} ,$$

where *d* is the particle diameter (m), *γ*'_s_ is the unit weight of the soil particle (kN/m^3^), *C*_L_ and *C*_D_ are the coefficients (dimensionless) of uplift force and drag force, respectively, *ρ*_w_ is the density of water (kg/m^3^), and *u*_b_ is the velocity (m/s) of the water flow near the slope surface.

The seepage force can be expressed as:

5 $$F_{{\text{P}}} = \frac{{\uppi }}{{6\left( {1 - n} \right)}}\gamma_{{\text{w}}} d^{3} \sin \theta ,$$

where *n* is the porosity of soil (dimensionless).

For cohesive particles, it can be considered that cohesion is generated by the contact of film water around the particles, and the expression is written as^23^:

6 $$F_{{\text{c}}} = \frac{{\uppi }}{2}q_{0} d\delta_{0}^{3} \left( {\frac{1}{{m^{2} }} - \frac{1}{{\delta_{1}^{2} }}} \right),$$

where *q*_0_ is the bonding force per unit area, with *q*_0_ = 1.3 × 10^10^ N/m^2^; *δ*_0_ is the diameter of a water molecule, with *δ*_0_ = 3 × 10^−10^ m; *δ*_1_ is the thickness of the film water, with *δ*_1_ = 4 × 10^−7^ m; and *m* is half of the closest gap distance between the particles, which can be calculated by Eq. (7)^23^.

7 $$\frac{{\gamma^{\prime}_{{\text{s}}} }}{{\gamma_{{\text{s}}} }} = \frac{2}{9}{\uppi }\left( {1 - \frac{m}{{4\delta_{1} }}} \right)\left( {\frac{d}{d + 2m}} \right)^{3} ,$$

where *γ*_s_ is the unit weight of soil (kN/m^3^).

Due to the equilibrium of forces acting normal to the slope surface, the normal force *F*_N_ can be expressed as:

8 $$F_{{\text{N}}} = F_{{\text{G}}} + F_{{\text{c}}} - F_{{\text{L}}} = \frac{{\uppi }}{6}\left( {\gamma^{\prime}_{{\text{s}}} - \gamma_{{\text{w}}} } \right)d^{3} \cos \theta + \frac{{\uppi }}{2}q_{0} d\delta_{0}^{3} \left( {\frac{1}{{m^{2} }} - \frac{1}{{\delta_{1}^{2} }}} \right) - \frac{{\uppi }}{8}C_{{\text{L}}} \rho_{{\text{w}}} d^{2} u_{{\text{b}}}^{2} .$$

Therefore, the friction force *F*_f_ can be written as Eq. (9) by introducing Eq. (8).

9 $$F_{{\text{f}}} = F_{{\text{N}}} \tan \varphi = \left[ {\frac{{\uppi }}{6}\left( {\gamma^{\prime}_{{\text{s}}} - \gamma_{{\text{w}}} } \right)d^{3} \cos \theta + \frac{{\uppi }}{2}q_{0} d\delta_{0}^{3} \left( {\frac{1}{{m^{2} }} - \frac{1}{{\delta_{1}^{2} }}} \right) - \frac{{\uppi }}{8}C_{{\text{L}}} \rho_{{\text{w}}} d^{2} u_{{\text{b}}}^{2} } \right] \cdot \tan \varphi ,$$

where *φ* is the friction angle of the soil (°).

In this context, sliding failure is adopted to describe the incipient slope soil motion. When the sliding force is equal to the resistance, the soil particles are in a critical state (see Eq. 10).

10 $$F_{{\text{f}}} = F_{{\text{D}}} + F_{{\text{P}}} + F_{{\text{G}}} \sin \theta .$$

We substitute Eqs. (2), (4), (5), and (9) into Eq. (10), and then the expression for the critical surface shear velocity *u*_bc_ can be written as:

11 $$u_{{{\text{bc}}}} = \sqrt {\frac{{12\left( {1 - n} \right)\alpha \tan \varphi + 4gd^{2} \left[ {\left( {G_{{\text{s}}} - 1} \right)\left( {1 - n} \right)\left( {\cos \theta \tan \varphi - \sin \theta } \right) - \sin \theta } \right]}}{{3d\left( {1 - n} \right)\left( {C_{{\text{D}}} + C_{{\text{L}}} \tan \varphi } \right)}}} ,$$

where *G*_s_ is the specific gravity of the soil particle, with *G*_s_ = *γ'*_s_/*γ*_w_; *γ*_w_ is the unit weight of water (kN/m^3^); *α* is an integrated parameter (m^3^/s^2^), with $\alpha = {q_0}{\delta_0^3}({1/m^2}-{1/{\delta_1^2}})/\rho_w$.

The friction velocity u_*_ is empirically related to the surface shear velocity *u*_b_ and their relation is written as^24^:

12 $$u_{{\text{b}}} = \beta u_{*} ,$$

where *β* is an empirical parameter whose value varies between 5.6 and 8.51. Herein, *β* = 5.6.

Furthermore, the surface shear stress *τ*_a_ is related to the friction velocity u_*_, which is expressed as^25^:

13 $$\tau_{{\text{a}}} = \rho_{{\text{w}}} u_{*}^{2} .$$

Thus, the parameters in the critical state can be expressed as follows:

14 $$\left\{ \begin{gathered} u_{{*{\text{c}}}} = \frac{1}{\beta }u_{{{\text{bc}}}} \hfill \\ \tau_{{\text{c}}} = \rho_{{\text{w}}} u_{{\text{*c}}}^{2} , \hfill \\ \end{gathered} \right.$$

where u_*c_ is the critical friction velocity (m/s) and *τ*_c_ is the critical surface shear stress (Pa).

Combining Eq. (11) with Eq. (14), the critical surface shear stress *τ*_c_ can be given by the following equation:

15 $$\tau_{{\text{c}}} = \frac{{3\left( {1 - n} \right)\alpha \tan \varphi + gd^{2} \left[ {\left( {G_{{\text{s}}} - 1} \right)\left( {1 - n} \right)\left( {\cos \theta \tan \varphi - \sin \theta } \right) - \sin \theta } \right]}}{{0.0235d\left( {1 - n} \right)\left( {C_{{\text{D}}} + C_{{\text{L}}} \tan \varphi } \right)}}.$$

This formula can be employed to estimate the critical shear stress for the incipient motion of the soil.

#### Excess shear stress equation

According to Eq. (1), the excess shear stress equation can be rewritten as follows:

16 $$\varepsilon = k_{{\text{d}}} \left( {\tau_{{\text{a}}} - \tau_{{\text{c}}} } \right).$$

In this formula, the erosion rate *ε* and the surface shear stress *τ*_a_ are the parameters that need to be measured. In this way, the erodibility coefficient *k*_d_ and the critical shear stress *τ*_c_ of the soil can be obtained based on linear regression^26^.

In addition, the erosion rate *ε* is easy to measure, as it is defined by:

17 $$\varepsilon = \frac{M}{St},$$

where *M* is the weight (kg) of sediment washed away, *t* is the scouring time (s), and *S* is the area of the eroded zone (m^2^).

**Figure 2:**
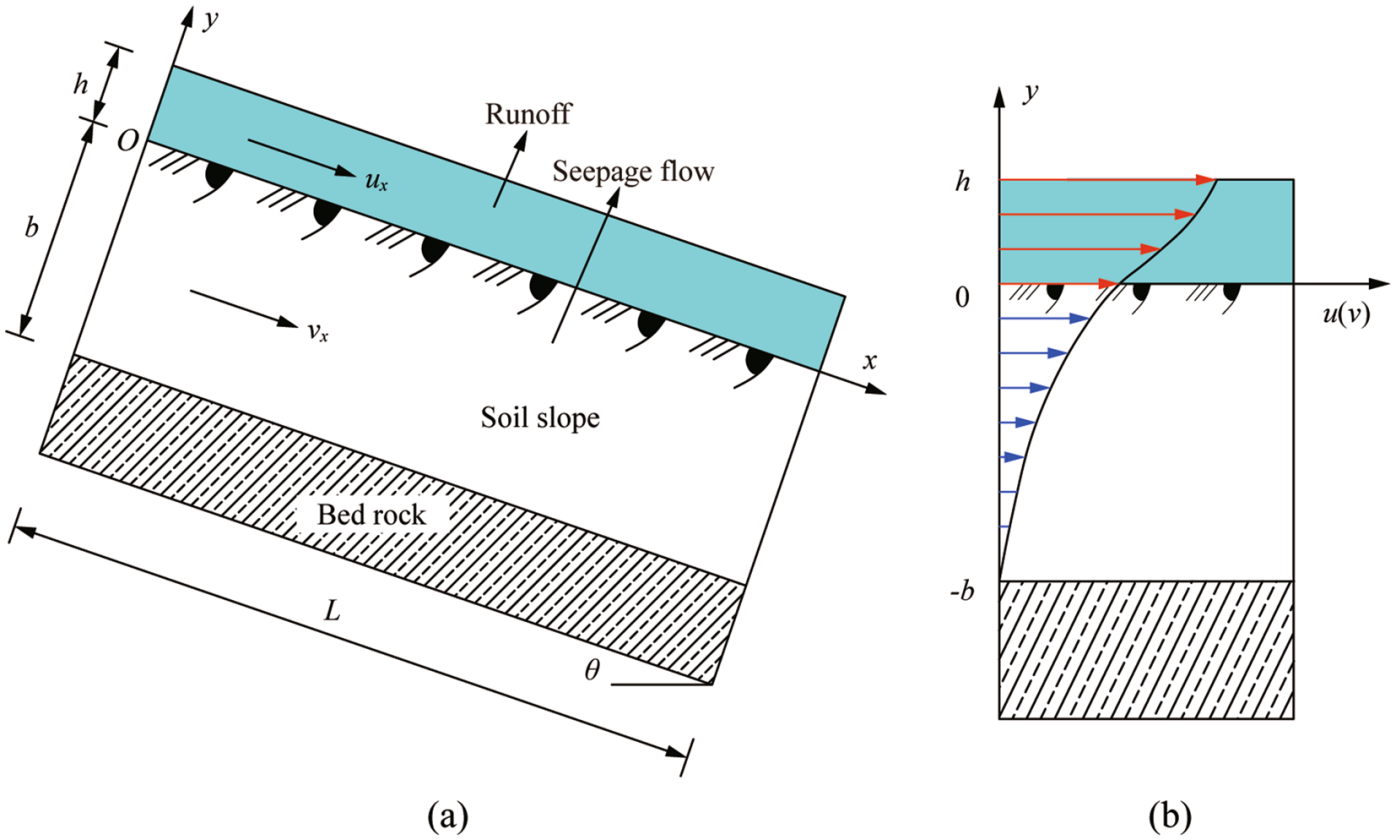
Coupled runoff and seepage in the soil slope model and sketch of flow velocity distribution in the *y* direction.

Moreover, it is vital to determine the surface shear stress *τ*_a_ and have it measured. Herein, we modeled a simplified soil slope (Fig. 2a) and considered runoff-seepage coupling^18^. The soil slope has a slope gradient of *θ*, and the soil properties include porosity *n* and permeability *K*. In this model, the motions of runoff and seepage are described by the Navier–Stokes equation and the Brinkman-extended Darcy equation, respectively. The runoff and seepage velocities are denoted as *u* and *v*, respectively, and *b* denotes the thickness of the soil layer. In addition, there is impermeable bedrock under the soil layer.

Yuan et al.^18^ derived the velocity distribution in the *y* direction, which is expressed as Eq. (18), and the velocity distribution is shown in Fig. 2b.

18 $$\left\{ {\begin{array}{*{20}l} {u = \frac{{\gamma_{{\text{w}}} \left( {\sin \theta + J} \right)}}{\eta }\left[ { - \frac{1}{2}y^{2} + hy + h\sqrt {nK} + K} \right]} \hfill & {(0 \le y \le h)} \hfill \\ {v = \frac{{\gamma_{{\text{w}}} \left( {\sin \theta + J} \right)}}{\eta }\left[ {\frac{{h\sqrt {nK} - K{\text{e}}^{{ - b\sqrt {{n \mathord{\left/ {\vphantom {n K}} \right. \kern-\nulldelimiterspace} K}} }} }}{{{\text{e}}^{{ - 2b\sqrt {{n \mathord{\left/ {\vphantom {n K}} \right. \kern-\nulldelimiterspace} K}} }} + 1}} \cdot {\text{e}}^{{y\sqrt {{n \mathord{\left/ {\vphantom {n K}} \right. \kern-\nulldelimiterspace} K}} }} - \frac{{h\sqrt {nK} + K{\text{e}}^{{b\sqrt {{n \mathord{\left/ {\vphantom {n K}} \right. \kern-\nulldelimiterspace} K}} }} }}{{{\text{e}}^{{2b\sqrt {{n \mathord{\left/ {\vphantom {n K}} \right. \kern-\nulldelimiterspace} K}} }} + 1}} \cdot {\text{e}}^{{ - y\sqrt {{n \mathord{\left/ {\vphantom {n K}} \right. \kern-\nulldelimiterspace} K}} }} + K} \right]} \hfill & {( - b \le y \le 0),} \hfill \\ \end{array} } \right.$$

where *u* and *v* are the velocities of runoff and seepage (m/s), respectively; *θ* is the slope gradient (°); *h* is the depth of runoff (m); *b* is the thickness of the soil layer (m); *n* and *K* are the porosity and permeability of the soil (m^2^), respectively; and *J* is the hydraulic gradient, with *J* = sin *θ*.

Then, according to Newton’s law of friction, the expression of surface shear stress can be derived as:

19 $$\tau_{{\text{a}}} = \eta \left. {\frac{{{\text{d}}u_{x} }}{{{\text{d}}y}}} \right|_{y = 0} = \gamma_{{\text{w}}} h\left( {\sin \theta + J} \right) = 2\gamma_{{\text{w}}} hJ.$$

In general, the average velocity *V* of the runoff cross-section is used to describe the incipient sediment motion, which is given by Manning’s equation as follows:

20 $$V = \frac{1}{\lambda }J^{\frac{1}{2}} R^{\frac{2}{3}} ,$$

where *V* is the average velocity (m/s) of the runoff cross-section; *λ* is Manning’s coefficient, which is used to indicate the slope roughness; and *R* is the hydraulic radius (m) of the runoff in the testing flume, which is approximately equal to the runoff depth *h*.

According to Eq. (20), the discharge per unit width of runoff can be written as:

21 $$q = Vh = \frac{1}{\lambda }\left( {\sin \theta } \right)^{\frac{1}{2}} h^{\frac{5}{3}} ,$$

where *q* is the discharge per unit width (m^2^/s).

Combining Eq. (19) with Eq. (21), we can obtain an alternative expression for the surface shear stress:

22 $$\tau_{{\text{a}}} = 2\gamma_{{\text{w}}} \lambda^{\frac{3}{5}} q^{\frac{3}{5}} \left( {\sin \theta } \right)^{\frac{7}{10}} .$$

Therefore, in laboratory tests, the surface shear stress *τ*_a_ can be measured indirectly when the discharge per unit width is known. According to a series of scouring tests, a fitted line can be obtained, resulting in the *k*_d_ and *τ*_c_ -values. Furthermore, the obtained *τ*_c_-values lack physical significance.

### Experiment

#### Experimental device

**Figure 3:**
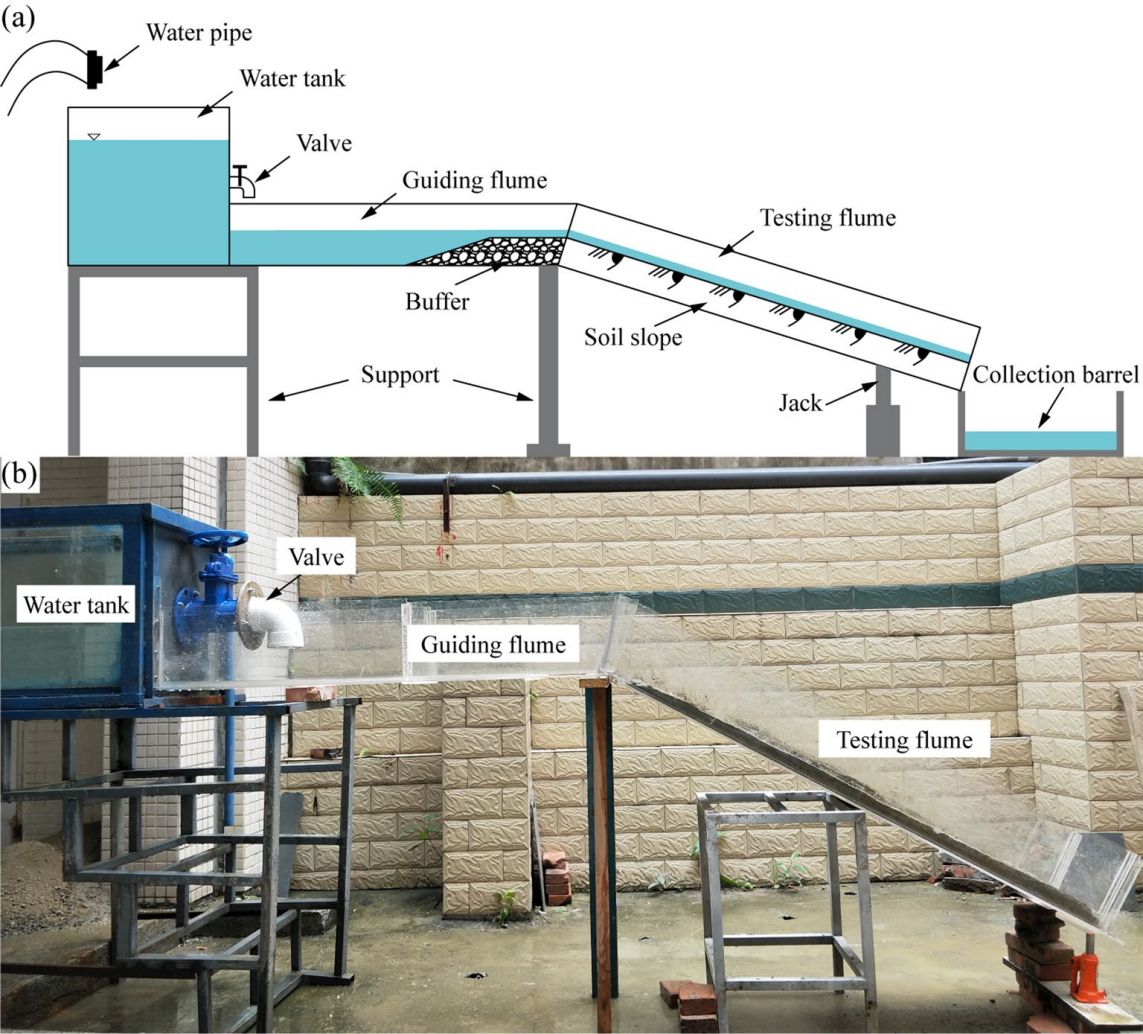
Diagram of the scouring experimental device.

The scouring device (Fig. 3) mainly consists of a water tank, a guiding flume, a testing flume, and a collection barrel.

- Water tank: This tank is used to provide a constant water difference. A valve set to adjust the flux is provided on the outfall side of the water tank.
- Guiding flume: The size of the guiding flume is 1.4 m × 0.35 m × 0.25 m (length × width × height). A reverse filter is laid at the end of the guiding flume to slow down the runoff and ensure laminar motion.
- Testing flume: The size of the testing flume is 1.7 m × 0.35 m × 0.25 m (length × width × height). The testing flume is used to place the testing material and observe the scouring process. A bolted connection exists between the guiding flume and the testing flume, which allows the slope gradient to be adjusted by changing the height of the jack to rotate the testing flume.
- Collection barrel: This barrel is used to collect the discharged water and the sediment washed away. The collected water and sediment are weighed in a certain period to obtain the discharge per unit width and the scouring rate.

#### Materials and parameters

**Figure 4:**
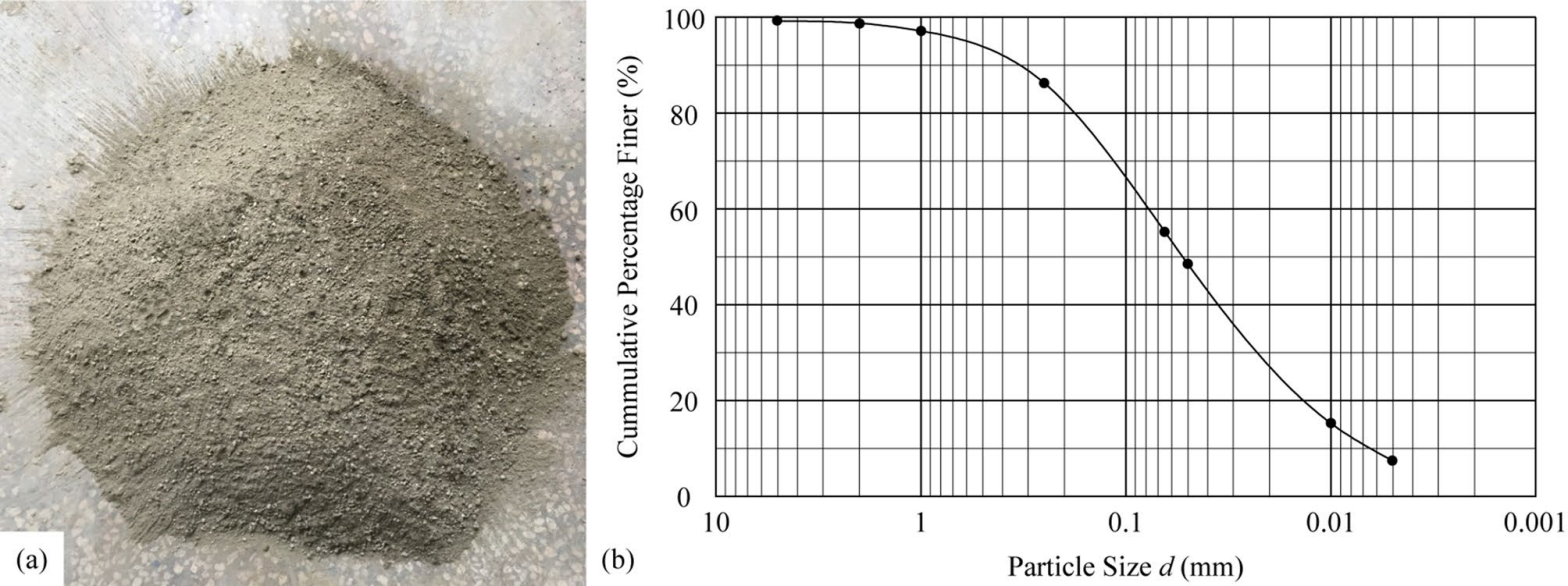
(**a**) Soil material used in the scouring experiment; (**b**) grading curve of the soil material.

The testing material was taken from a highway slope site on Chengdu Third Ring Road, China. The soil material object after drying and smashing is shown in Fig. 4a, and the grading curve is shown in Fig. 4b.

In addition, the physical parameters of the material and other related parameters can be found in Table 1.

**Table 1:** Parameters used in the calculation of Eq. (16).

Parameter	Unit weight of soil *γ*_s_ (kN/m^3^)	Unit weight of particle *γ'*_s_ (kN/m^3^)	Unit weight of water *γ*_w_ (kN/m^3^)	Porosity of soil *n*	Internal friction angle of soil *φ* (°)
Value	18.2	26.5	10.0	0.43	30.6
Parameter	Median diameter of soil *d*_50_ (mm)	*α* (m^3^/s^2^)	Coefficient of uplift force *C*_L_	Coefficient of drag force *C*_D_	Manning’s coefficient *λ*
Value	0.052	1.75 × 10^−6^	0.1	0.4	0.015

#### Testing process

First, the flow rate of the valve should be calibrated by marking the valve position for a certain flow rate (0.4 L/s, 0.5 L/s and 0.6 L/s). Second, the soil material should be filled in the testing flume with the flat slope surface parallel to the flume surface. Then, the desired slope gradient can be adjusted through the length of the jack. Before the scouring experiment begins, a low-discharge flow should be ensured and infiltrates into the soil layer, which will stop when runoff from the slope surface merely begins to form. The scouring process will last for 10 min, but the experiment will end if the slope fails.

During the scouring process, the collection barrel is replaced every 2 min. Finally, when the scouring process is finished, the scoured sediment collected in the barrel is dried and then weighed. Using the weight of the sediment *M*, the scouring rate can be calculated by Eq. (17); herein, *S* = 0.595 m^2^. Moreover, the orthogonal test should be conducted under three levels of flow rate (0.4 L/s, 0.5 L/s and 0.6 L/s) and four levels of slope gradient (26.6°, 29.7°, 33.7° and 38.7°).

## Results and discussion

### Test results

Following the above testing steps, we obtained a series of scouring data. The weights of soil washed away under different conditions of flow rate and slope gradient at *t* = 2, 4, 6, 8 and 10 min are shown in Table 2.

**Table 2:** Accumulated weight of soil washed away at *t* = 2, 4, 6, 8 and 10 min.

Slope gradient *θ* (°)	Flow flux *Q* (L/s)	Accumulated weight of soil washed away at different time *M* (kg)				
		*t* = 2 min	*t* = 4 min	*t* = 6 min	*t* = 8 min	*t* = 10 min
26.6	0.4	0.128	0.266	0.408	0.523	0.644
	0.5	0.155	0.326	0.498	0.652	0.798
	0.6	0.226	0.472	0.723	0.960	1.182
29.7	0.4	0.164	0.350	0.521	0.679	0.830
	0.5	0.215	0.436	0.65	0.838	1.002
	0.6	0.272	0.557	0.832	1.098	1.350
33.7	0.4	0.186	0.382	0.595	0.79	0.978
	0.5	0.226	0.482	0.722	0.944	1.155
	0.6	0.302	0.620	0.941	1.256	1.557
38.7	0.4	0.226	0.458	0.681	0.898	1.106
	0.5	0.281	0.584	0.901	1.195	–
	0.6	0.375	0.772	1.160	–	–

**Figure 5:**
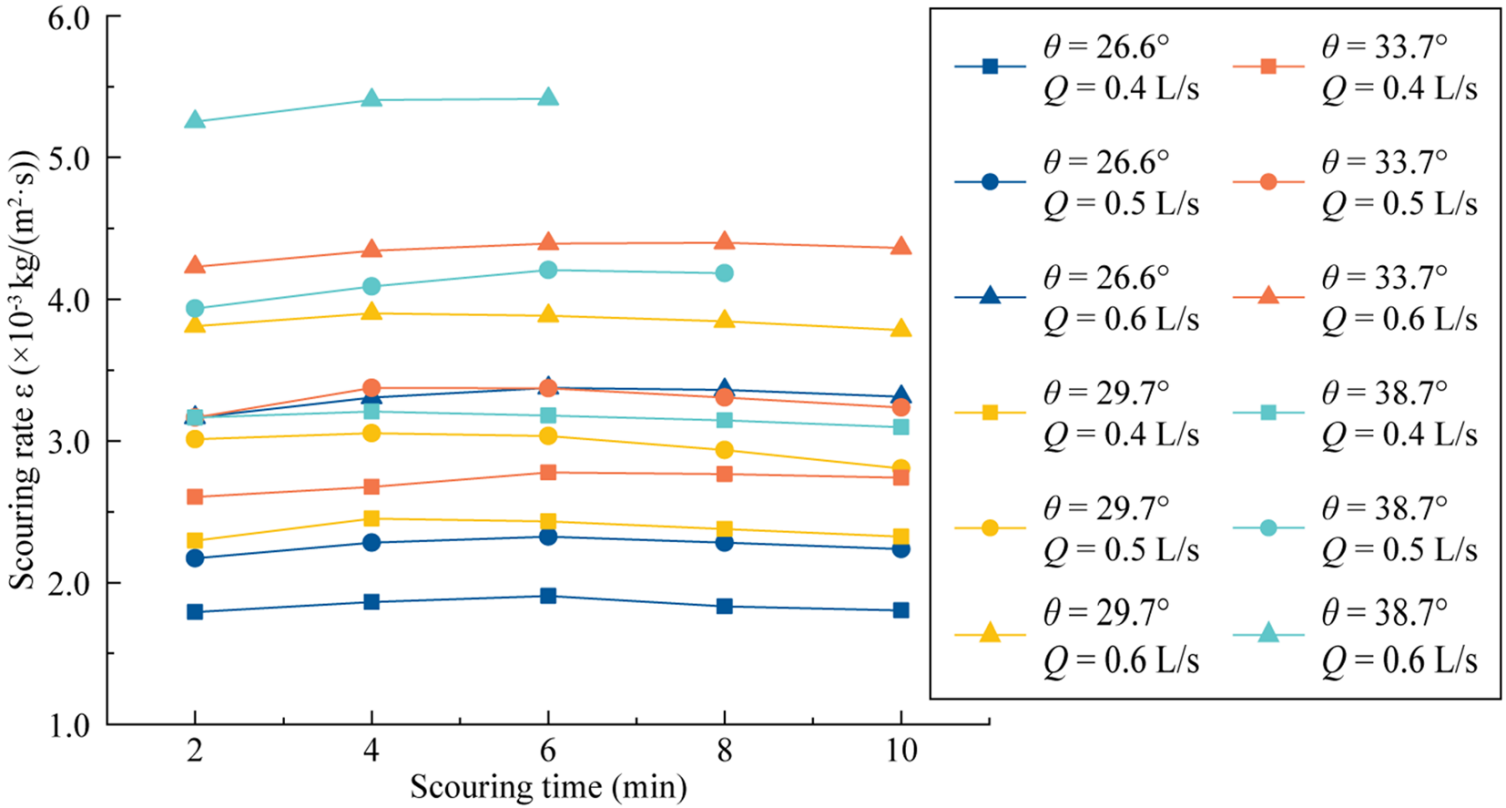
Variation in the erosion rate under different slope gradients and flow rates.

According to Eq. (17), the erosion rate *ε* can be calculated for different times *t* (Fig. 5). For each curve, the *ε*-values varied slightly with time, indicating that the slope was steady. Thus, we adopted the average *ε*-value of the curves to represent the erosion rate of the scouring process.

**Figure 6:**
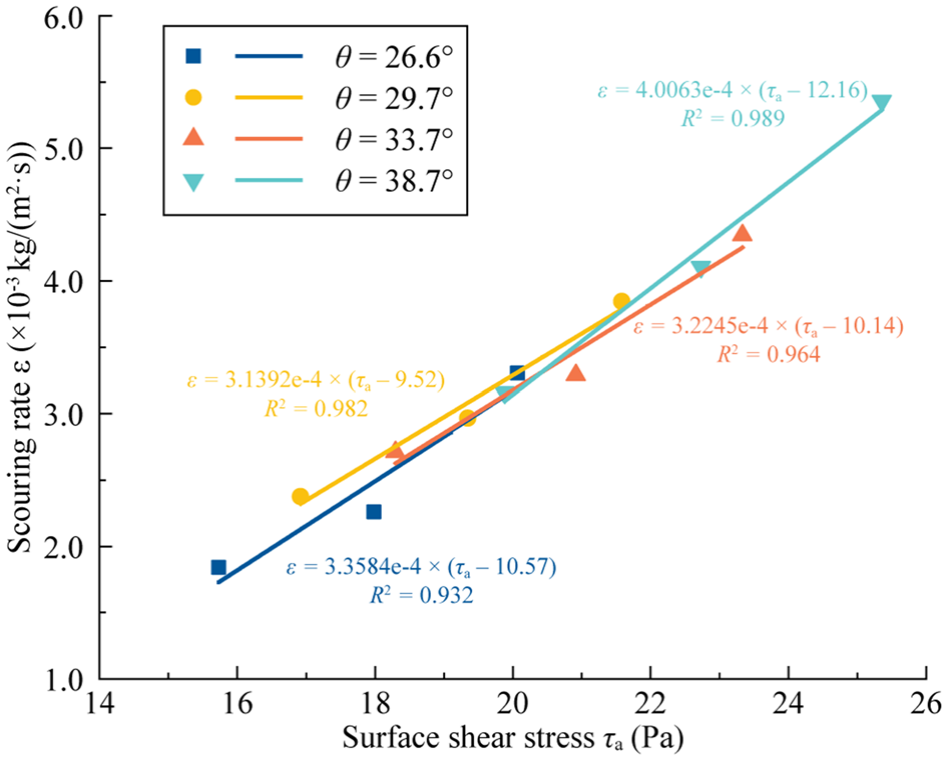
Linear relationship between the scouring rate and shear stress.

The four fitted lines (Fig. 6) were obtained through a series of *ε − τ*_a_ points categorized by the slope gradient. These lines contain the coefficient values of the excess shear stress equation, as derived in Table 3.

**Table 3:** Coefficient conclusion of the fitting lines and comparison between the fitted and theoretical critical shear stress values.

Slope gradient *θ* (°)	Fitting values from parameters		Theoretical values of critical shear stress *τ*_c,t_ (Pa)	Relative error (%)
	Erodibility coefficient *k*_d_ (× 10^−4^ s/m)	Critical shear stress *τ*_c,e_ (Pa)		
26.6	3.3584	10.57	9.33	11.73
29.7	3.1392	9.52	9.32	2.10
33.7	3.2245	10.14	9.31	8.19
38.7	4.0063	12.16	9.30	23.52

By comparison, theoretical values of the critical shear stress are calculated from Eq. (15), which are also given in Table 3. The fitted results for the critical shear stress have small relative errors (< 15%) with the theoretical results for *θ* = 26.6°, 29.7° and 33.7°, while the relative error when *θ* = 38.7° is much larger than 20%.

### Discussion

Studies on the critical shear stress for soil erosion have focused on the direct measurement of critical shear stress. We avoided complex equipment and unquantifiable criteria for the critical shear stress and found this simple method to determine the critical shear stress by simply measuring the weight of soil particles being washed away and the water discharge. This method is discussed as follows.

#### Regarding the testing results

**Figure 7:**
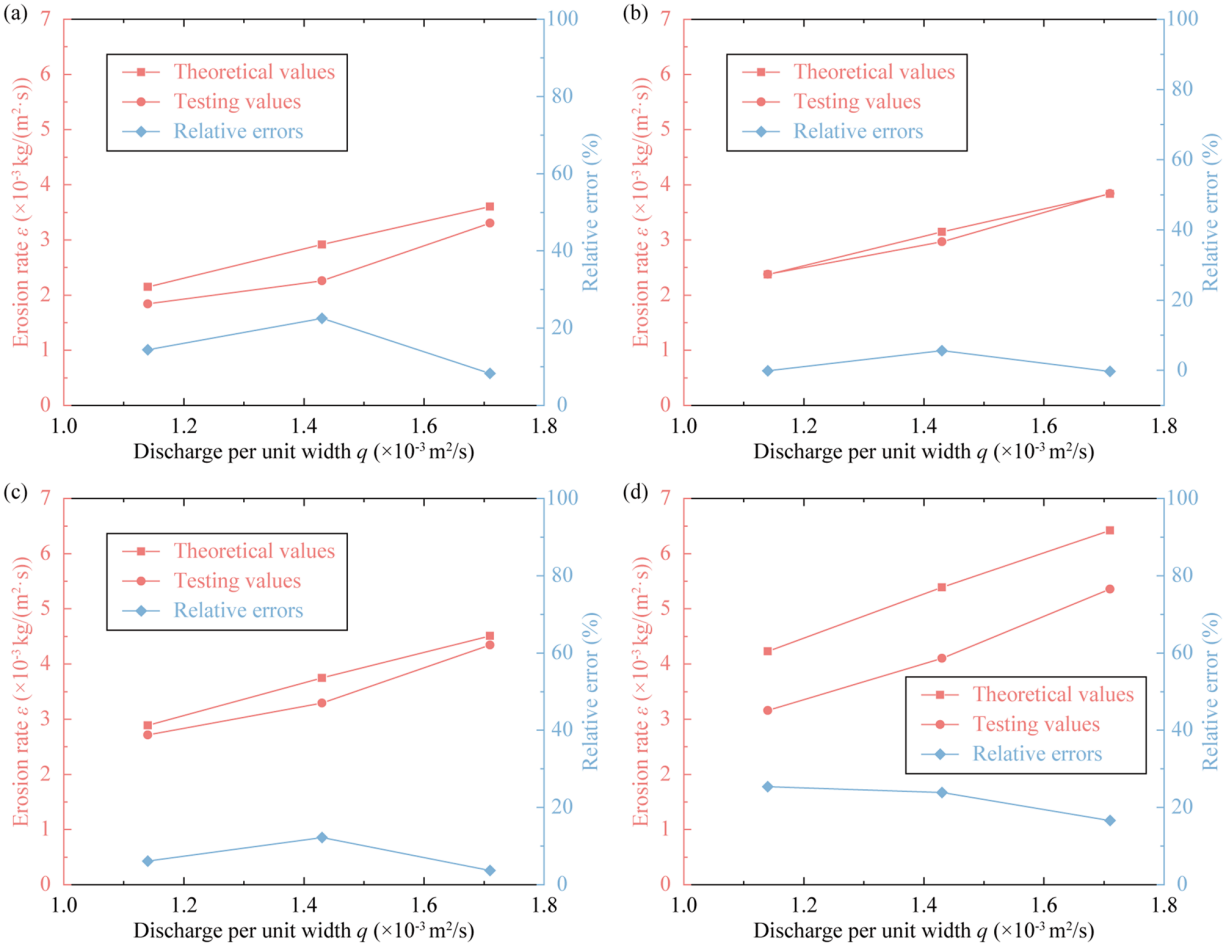
Comparison of theoretical and testing values of the erosion rate under different slope gradients: (**a**) *θ* = 26.6°, (**b**) *θ* = 29.7°, (**c**) *θ* = 33.7°, and (**d**) *θ* = 38.7°.

The indicator *ε*, which can be calculated using Eq. (16), was applied to certify the accuracy of the simple measurement. Here, the erodibility coefficient *k*_d_ adopts the fitted values for the corresponding slope gradient, while the critical shear stress *τ*_c_ adopts the theoretical results of Eq. (15). The calculated results were compared with the testing results in Fig. 7.

The results indicate that the slope gradient and the flux have a positive effect on the erosion rate *ε*. Taking the calculated results as an example, when the slope gradient is 26.6°, the result with a flux of 0.5 L/s is 1.36 times greater than that with a flux of 0.4 L/s; when the flux is 0.5 L/s, the result with a slope gradient of 29.7° is 1.08 times greater than that with a slope gradient of 26.6°. In addition, it was found that the theoretical results obtained from Eq. (16) are larger than the testing results.

In the analysis of the results, most of the error results were found to be less than 15%, indicating a reliable estimation through this measurement. However, when *θ* = 38.7°, there is a relatively large difference between the fitted and theoretical *τ*_c_-values, with a relative error of more than 20%, and the *ε*-values have a similar error result. This may be due to different flow patterns, as laminar flow is assumed in the runoff-seepage coupled model, but the flow motion is actually turbulent. However, the erodibility coefficient *k*_d_ in erosion rate estimation needs to be measured by scouring tests.

#### Regarding the comparison with published formulas

**Figure 8:**
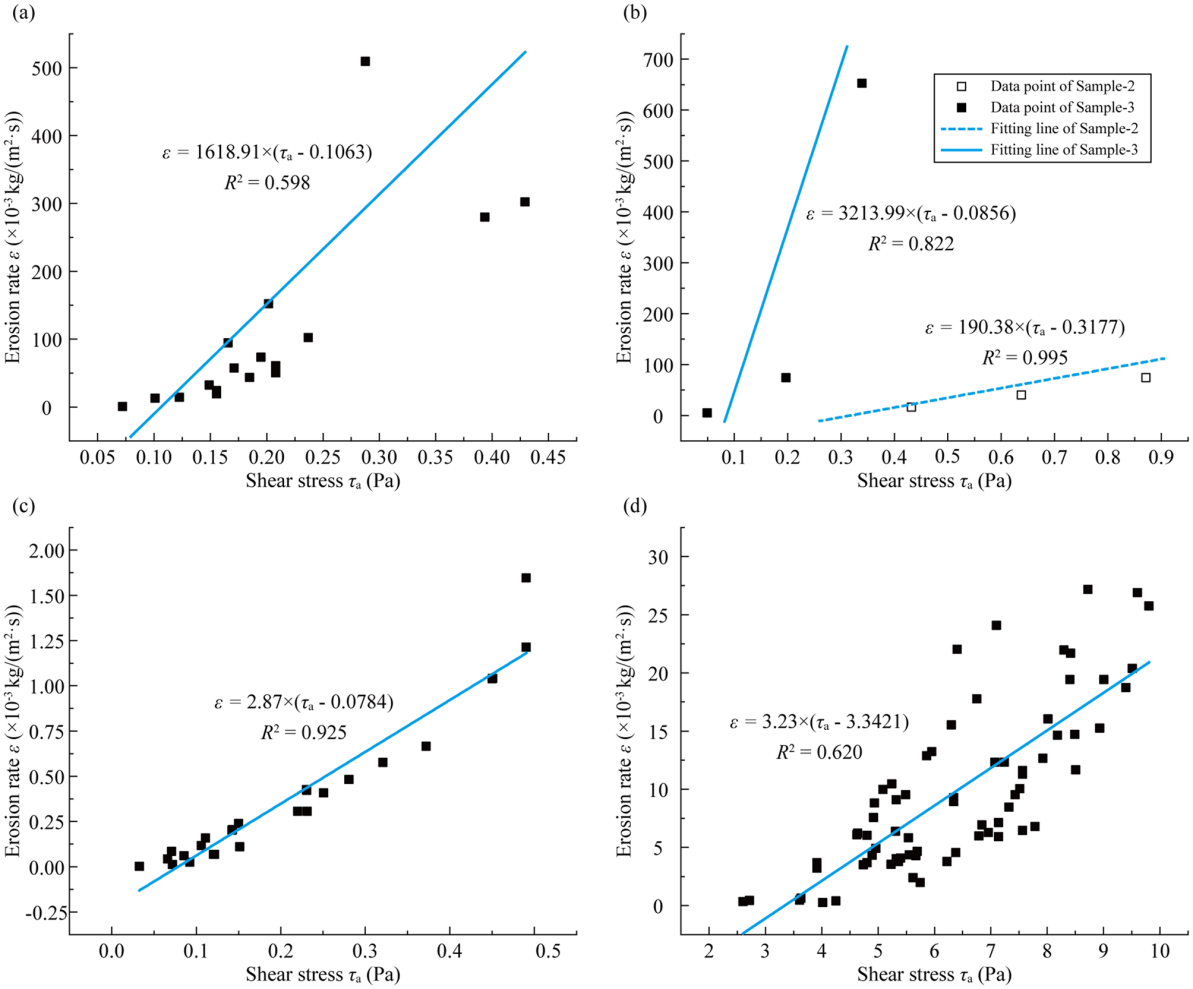
Collected *ε − τ*_c_ data points and the regression lines: (**a**) Sample-1, (**b**) Sample-2 and Sample-3, (**c**) Sample-4, and (**d**) Sample-5.

To validate whether Eq. (15) can be widely used in other references, test data from the reported articles were collected (see Table 4). The experimental data from these references are shown in Fig. 8.

**Table 4:** Fundamental physical parameter values collected from different references.

No	Materials	Median diameter *d*_50_ (mm)	Unit weight *γ*_s_ (kN/m^3^)	Internal friction angle *φ* (°)	References
Sample-1	Sand	0.74	18.0	10.6	Kwon et al.^27^
Sample-2	Soft marine soil	0.018	17.8	4.23	Kwon et al.^28^
Sample-3	Silica sand	0.32	18.0	15	Kwon et al.^28^
Sample-4	Soil	0.06	17.2	30	Ravens and Gschwend^29^
Sample-5	Soil	0.075	18.3	30	Gilley et al.^30^

Through linear regression, we obtained the critical shear stress and the erodibility coefficient of the five samples in Table 5. In addition, the *τ*_c_-values calculated by Eq. (15) are also given in Table 5.

**Table 5:** Erodibility coefficient and critical shear stress obtained by linear regression and comparison between the fitting critical shear stress values and the calculation results from Eq. (15).

No	Results obtained by linear regression		Critical shear stress calculated by Eq. (15) (Pa)	Relative error (%)	Absolute error (Pa)
	Erodibility coefficient *k*_d_ (× 10^−3^ s/m)	Critical shear stress *τ*_c_ (Pa)			
Sample-1	1618.91	0.1063	0.1250	− 14.96	− 0.0187
Sample-2	190.38	0.3177	0.2810	13.06	0.0367
Sample-3	3213.99	0.0856	0.1162	− 26.33	− 0.0306
Sample-4	2.87	0.0784	0.1151	− 31.89	− 0.0367
Sample-5	3.23	3.3421	3.2095	4.13	0.1326

By comparison, Eq. (15) also shows advantages in accurately estimating the critical shear stress of soils with a maximum relative error value of 31.89% and a maximum absolute error value of 0.1326 Pa.

Moreover, we adopted the following two empirical formulas for comparison purposes^31^:

23 $$\tau_{{\text{c}}} = 3.54 \times 10^{{ - 28.1d_{50} }} ,$$

24 $$\tau_{{\text{c}}} = 0.493 \times 10^{{0.0182P_{{\text{c}}} }} ,$$

where *d*_50_ is the mean diameter of the soil (m), and *P*_c_ is the percentage of clay by weight (%).

Furthermore, we employed the test data obtained by Lei et al.^32^. The soil sample was a typical silt–clay soil from the Loess Plateau of China. The clay particles, whose size is less than 0.01 mm, account for approximately 56% of the weight. Table 6 presents the *τ*_c_-values for different slope gradients from Lei et al.^32^ and the calculated results of Eqs. (15), (23) and (24).

**Table 6:** Comparison of critical shear stress results calculated by Eqs. (15), (23) and (24).

Slope gradient (°)	Experimental *τ*_c_-value (Pa)	*τ*_c_-value by Eq. (15) (Pa)	Relative error (%)	*τ*_c_-value by Eq. (23) (Pa)	Relative error (%)	*τ*_c_-value by Eq. (24) (Pa)	Relative error (%)
5	3.191	3.550	11.24	5.153	61.48	3.538	10.87
10	3.960	3.548	10.41	5.153	30.12	3.538	10.66
15	4.130	3.546	14.14	5.153	24.77	3.538	14.34
20	4.385	3.544	19.18	5.153	17.51	3.538	19.32
25	4.565	3.542	22.41	5.153	12.88	3.538	22.50

The *τ*_c_-values calculated from Eq. (15) were found to be the most consistent with the experimental *τ*_c_-values among the three equations. In addition, the calculated *τ*_c_-values from Eq. (15) vary with the slope gradient, while the results calculated by the other two equations do not. On the one hand, the last two formulas are incomplete, as they focus on only one parameter. On the other hand, both formulas are empirical and lack physical significance. Moreover, Eq. (15) is derived based on the force equilibrium of a single particle. In the derivation process, multiple parameters are considered, namely particle diameter *d*, porosity *n*, specific gravity *G*_s_, slope gradient *θ*, and internal friction angle *φ*. Therefore, it can be concluded that the critical formula (Eq. 15) derived in this paper is applicable to estimate the critical shear stress for the incipient particle motion of cohesive soil slopes.

#### Regarding sensitivity

According to Eq. (15), the critical shear stress *τ*_c_ is related to the particle diameter *d*, porosity *n*, soil unit weight *γ*_s_, slope gradient *θ*, internal friction angle *φ*, and integrated parameter of bonding force *α*. In addition, *α* is influenced by *d* and *γ*_s_ from Eqs. (6) and (7). Here, these parameters are discussed to determine their influences on the estimation of the critical shear stress *τ*_c_, and the parameter values are given in Table 7.

**Table 7:** Value range of the parameters for sensitivity analysis of the critical shear stress.

Parameters	Value range			
Soil particle diameter *d* (mm)	0.01–10			
Porosity *n*	0.25	0.30	0.35	0.40
Soil unit weight *γ*_s_ (kN/m^3^)	16.0	17.0	18.0	19.0
Slope gradient *θ* (°)	4	8	12	16
Internal friction angle *φ* (°)	25	30	35	40

**Figure 9:**
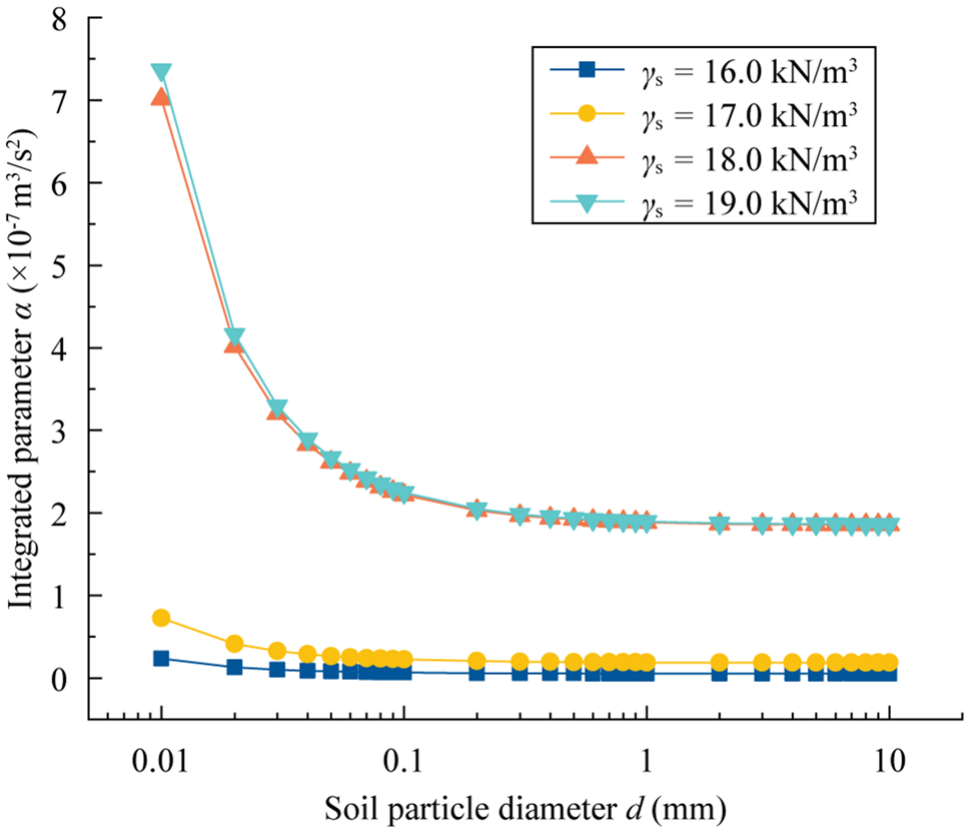
Integrated parameter of bonding force *α* varying with the soil unit weight and soil particle diameter.

Due to the influence of *d* and *γ*_s_ on the bonding force, the parameter *α* was first analyzed with the increase of *d*. The slope gradient *θ* = 10° and the values of other parameters can be found in Table 1. The results of *α* varying with *d* and *γ*_s_ can be seen in Fig. 9. Generally, *α* is negatively correlated with *d* and positively correlated with *γ*_s_. After *d* reaches 1 mm, the *α*-values no longer vary. Clearly, the *α*-curves show little difference when *γ*_s_ = 18.0 kN/m^3^ and *γ*_s_ = 19.0 kN/m^3^. When *d* equals 0.01 mm, the *α*-values are 0.2349 × 10^−7^ m^3^/s^2^, 0.7260 × 10^−7^ m^3^/s^2^, 7.0159 × 10^−7^ m^3^/s^2^, and 7.3638 × 10^−7^ m^3^/s^2^ with soil unit weights *γ*_s_ ranging from 16.0 kN/m^3^ to 19.0 kN/m^3^.

After the above discussion, the variation in the critical shear stress *τ*_c_ with *d* and *γ*_s_ can be further studied. Correspondingly, the *α*-curves should be used properly in the calculation process of *τ*_c_ with similar soil unit weights.

**Figure 10:**
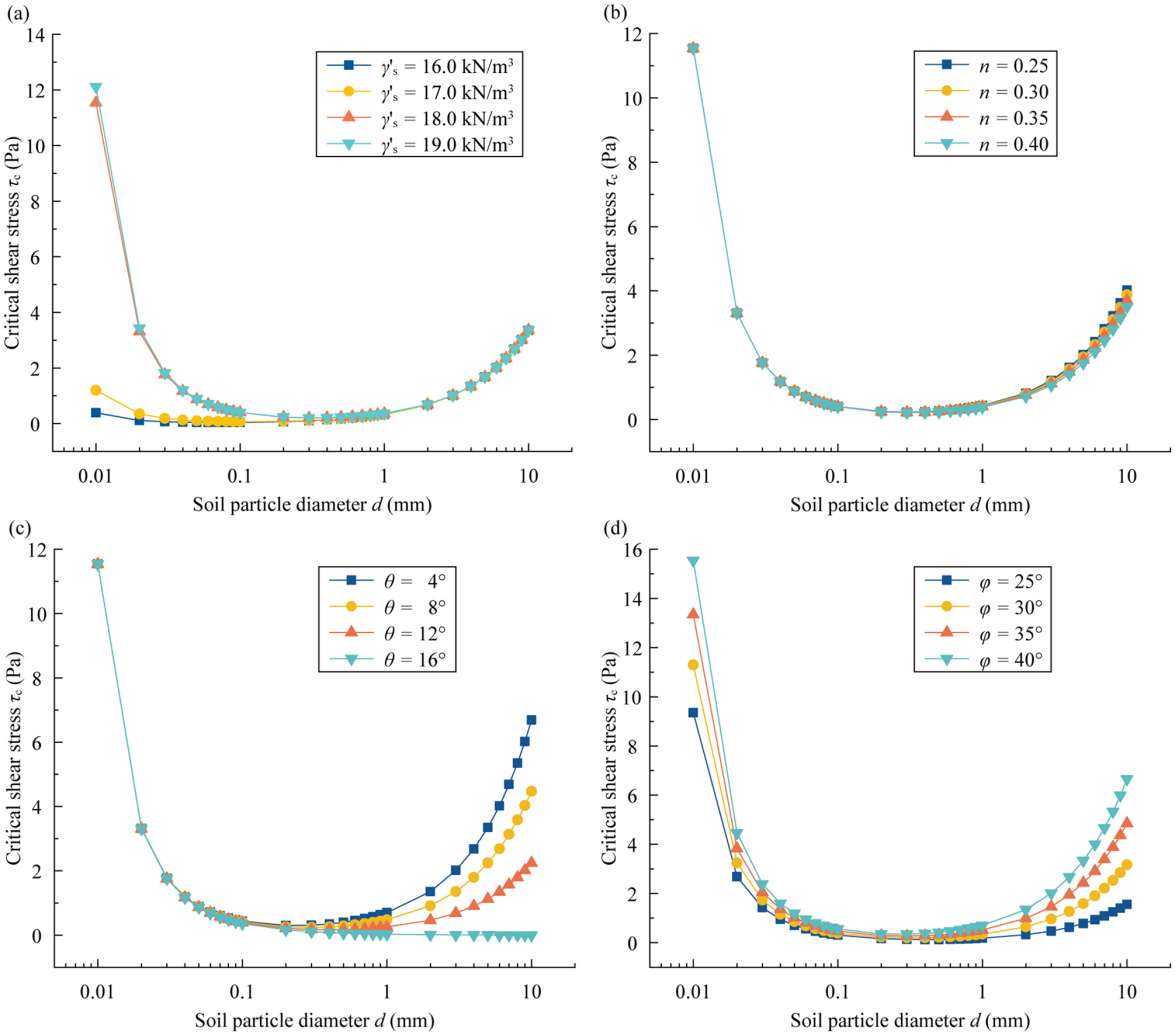
Sensitivity analysis for the effect of different parameters on the estimation of the critical shear stress: (**a**) soil unit weight, (**b**) porosity, (**c**) slope gradient, and (**d**) internal friction angle.

The analyzed results are plotted in Fig. 10a. *τ*_c_ is positively correlated with *γ*_s_, and it first decreases and then increases with increasing *d*. When *d* is less than 1 mm, the difference between the four curves is obvious. The critical shear stress *τ*_c_ decreases from 11.54 to 1.20 Pa with *d* = 0.01 mm when *γ*_s_ decreases from 18.0 kN/m^3^ to 17.0 kN/m^3^, and it decreases from 0.40 to 0.07 Pa with *d* = 0.1 mm. The decreasing gap of *τ*_c_ is enlarged with decreasing of soil particle diameter. Nevertheless, there is almost no difference between the four curves when *d* is larger than 1 mm. It could be inferred that the bonding force plays a vital role when *d* < 1 mm while it can be ignored when *d* ≥ 1 mm.

The sensitivity analysis of *τ*_c_ for the other three parameters (porosity *n*, slope gradient *θ*, and internal friction angle *φ*) is also plotted in Fig. 10b–d. The *α*-values refer to the *α*-curve in Fig. 9 with *γ*_s_ = 18.0 kN/m^3^.

In Fig. 10b, *τ*_c_ shows a slight difference with diverse porosity values, and the difference can be observed when *d* ≥ 1 mm. In addition, *τ*_c_ is negatively correlated with *n*. When *n* increases from 0.30 to 0.35, *τ*_c_ decreases from 3.88 to 3.70 Pa with *d* = 10 mm and decreases from 0.42 to 0.40 Pa with *d* = 1 mm. The variation difference is enlarged as *d* increases.

In Fig. 10c, *τ*_c_ can be obviously influenced by *θ* when *d* ≥ 0.1 mm, and *τ*_c_ is negatively correlated with *θ*. When *θ* increases from 8° to 12°, *τ*_c_ decreases from 0.41 to 0.38 Pa with *d* = 0.1 mm and from 0.48 to 0.26 Pa with *d* = 1 mm. Similarly, the variation difference is enlarged as *d* increases.

In Fig. 10d, *τ*_c_ can be obviously influenced by *φ* when *d* ranges from 0.01 to 10 mm, and *τ*_c_ is positively correlated with *φ*. When *φ* increases from 30° to 35°, *τ*_c_ increases from 11.30 to 13.35 Pa with *d* = 0.01 mm, from 0.23 to 0.29 Pa with *d* = 0.2 mm, and from 3.16 to 4.85 Pa with *d* = 10 mm. The variation difference first decreases and then increases with *d*.

According to the sensitivity analysis, the critical shear stress *τ*_c_ of slope cohesive soil is mainly influenced by the soil particle diameter *d*, internal friction angle *φ*, and soil unit weight *γ*_s_ while that of slope cohesionless soil is mainly influenced by the soil particle diameter *d*, internal friction angle *φ*, porosity *n* and slope gradient *θ*.

## Conclusions

In this paper, a formula has been derived for estimating the critical shear stress for the incipient particle motion of a cohesive soil slope. This formula is based on the force equilibrium of a single particle, which makes the derived formula physically significant.

To validate the derived formula, a series of scouring tests were conducted, and the excess shear stress equation was introduced to estimate the tested values of the critical shear stress. The results show that the calculated results from the derived formula are in good agreement with the tested results. In addition, two other formulas were employed for comparison with the derived formula. The derived formula shows great advantages in considering multiple parameters and has obvious physical significance.

The parameters influencing the critical shear stress were analyzed. The soil porosity, soil specific gravity and slope gradient have less influence on the critical shear stress. In addition, the critical shear stress is negatively influenced by the particle diameter and positively influenced by the internal friction angle.

## Supplementary Information

Supplementary Information.

## Data Availability

All data, models, and code generated or used during the study appear in the submitted article.
