# Supplementary material for: Estimating the critical shear stress for incipient particle motion of a cohesive soil slope
Source: Sci Rep. 2022 Jun 13;12:9736. doi: 10.1038/s41598-022-13307-w (PMC9192603; doi:10.1038/s41598-022-13307-w)
Supplement: Supplementary file 1 — Supplementary Information. [file 41598_2022_13307_MOESM1_ESM.docx]

# Appendix A: Derivation of Adhesive Force

The adhesive force *F*_B_ is generated by the contact of film water between adjacent soil particles. The water contact is shown in Fig. 11(a), and Ω is contact area whose vertical view is shown in Fig. 11(b). Firstly, *F*_B_ could be written as follows ^[23]^.

$F_{\text{B}}=\int_{\Omega} \frac{q_{0}\delta_{0}^{3}}{a^{3}} \text{d}\Omega$ (A1)

where *q*_0_ is the adhesive force of unit area, and normally *q*_0_ = 1.3×10^10^ N/m^2^; *δ*_0_ is diameter of water molecule, and *δ*_0_ = 3×10^-10^ m.


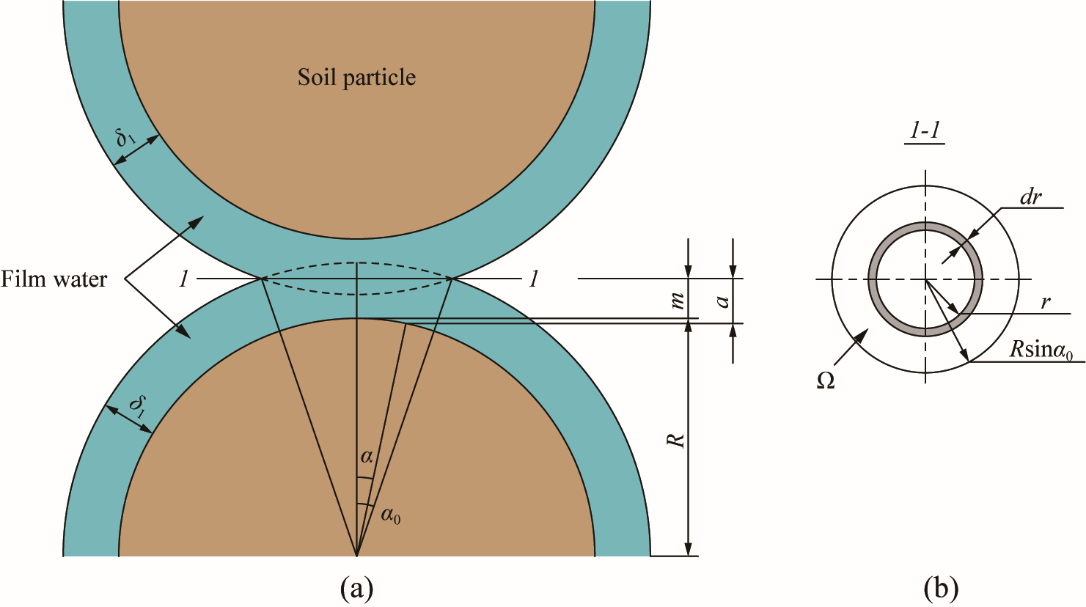


**Fig. 11.** (a) Sketch of film water contact between two soil particles, (b) Vertical view of film water contact area in 1-1' section

In Fig. 11(a), *m* means half of the closest gap distance between particles; *α* varies from 0 to *α*_0_; *δ*_1_ is the thickness of film water, and *δ*_1_ = 4×10^-7^ m. According to the geometric relationship, it could be found that *a* is related to *α*, and the relation could be described as,

$a=m+R\left(1-\cos\alpha\right)$ (A2)

In Fig. 11(b), it is known that the double integral could be solved in the form of polar coordinate, then it could be rewritten as,

$F_{\text{B}}=q_{0}\delta_{0}^{3}\int_{0}^{2\text{π}} \text{d}\theta\int_{0}^{\left(R+\delta_{1}\right)\sin\alpha_{0}} \frac{r}{\left[m+R\left(1-\cos\alpha\right)\right]^{3}} \text{d}r$ (A3)

where *r* is the radius of d*r* in area Ω and *r =* (*R* + *δ*_1_)·sin *α*.

Substituting the expression of *r* into Eq. (A3), then

$F_{\text{B}}=2\text{π}q_{0}\delta_{0}^{3}\int_{0}^{\alpha_{0}} \frac{\left(R+\delta_{1}\right)^{2}\sin\alpha\cos\alpha}{\left[m+R\left(1-\cos\alpha\right)\right]^{3}} \text{d}\alpha=2\text{π}q_{0}\delta_{0}^{3}\int_{\cos\alpha_{0}}^{1} \frac{\left(R+\delta_{1}\right)^{2}\cos\alpha}{\left[t_{0}+R\left(1-\cos\alpha\right)\right]^{3}} \text{d}\left(\cos\alpha\right)$ (A4)

In fact, *δ*_1_≪*R* and we supposed that *f =* cos *α*, then Eq. (A4) could be simplified as,

$F_{\text{B}}=2\text{π}q_{0}\delta_{0}^{3}\int_{\cos\alpha_{0}}^{1} \frac{R^{2}f}{\left[m+R\left(1-f\right)\right]^{3}} \text{d}f$ (A5)

Eq. (A5) could be solved by integration by parts as the following derivation,

$$F_{\text{B}}=2\text{π}q_{0}\delta_{0}^{3}\left\{\left.\frac{Rf}{2\left[m+R\left(1-f\right)\right]^{2}}\right|_{\cos\alpha_{0}}^{1}-\int_{\cos\alpha_{0}}^{1} \frac{R}{2\left[m+R\left(1-f\right)\right]^{2}} \text{d}f\right\}$$

$$=2\text{π}q_{0}\delta_{0}^{3}\left\{\left.\frac{Rf}{2\left[m+R\left(1-f\right)\right]^{2}}\right|_{\cos\alpha_{0}}^{1}-\left.\frac{1}{2\left[m+R\left(1-f\right)\right]}\right|_{\cos\alpha_{0}}^{1}\right\}$$

$$=2\text{π}q_{0}\delta_{0}^{3}\cdot\left.\frac{R\left(2f-1\right)-m}{2\left[m+R\left(1-f\right)\right]^{2}}\right|_{\cos\alpha_{0}}^{1}$$

$=2\text{π}q_{0}\delta_{0}^{3}\cdot\left\{\frac{R-m}{2m^{2}}-\frac{R\left(2\cos\alpha_{0}-1\right)-m}{2\left[m+R\left(1-\cos\alpha_{0}\right)\right]^{2}}\right\}$ (A6)

After deformation, Eq. (A6) could be rewritten as,

$$F_{\text{B}}=2\text{π}q_{0}\delta_{0}^{3}\cdot\left\{\left\{\frac{R+m}{2m^{2}}-\frac{R+m}{2\left[m+R\left(1-\cos\alpha_{0}\right)\right]^{2}}\right\}-\left\{\frac{1}{m}-\frac{m+R\left(1-\cos\alpha_{0}\right)}{\left[m+R\left(1-\cos\alpha_{0}\right)\right]^{2}}\right\}\right\}$$

$=2\text{π}q_{0}\delta_{0}^{3}\cdot\left\{\frac{R+m}{2}\left\{\frac{1}{m^{2}}-\frac{1}{\left[m+R\left(1-\cos\alpha_{0}\right)\right]^{2}}\right\}-\left[\frac{1}{m}-\frac{1}{m+R\left(1-\cos\alpha_{0}\right)}\right]\right\}$ (A7)

As *t*_0_ ≪ *R*, *δ*_1_ ≪ *R* and *δ*_1_ *= m* *+ R*(1 – cos *α*_0_), it could be simplified as,

$F_{\text{B}}=\text{π}q_{0}R\delta_{0}^{3}\cdot\left(\frac{1}{m^{2}}-\frac{1}{\delta_{1}^{2}}\right)$ (A8)

# Appendix B: Derivation of Constant *m*

The constant *m* is correlated to the unit weight of soil *γ'*_s_ and the particle arrangement. It is assumed that particle arrangement is shown in Fig. 12(a). In the *xOy* plane, shape of the zone occupied by a single particle is a parallelogram. The length in *x* direction of the parallelogram is *d* + 2*m*, and the length in *y* direction is $\frac{\sqrt{3}}{2}(d+2m)$. Similarly, the length in *z* direction is $\frac{\sqrt{3}}{2}(d+2m)$ as well in *xOz* plane. Therefore, volume of the zone occupied by the single particle is 3(*d* + 2*m*)^3^ / 4.


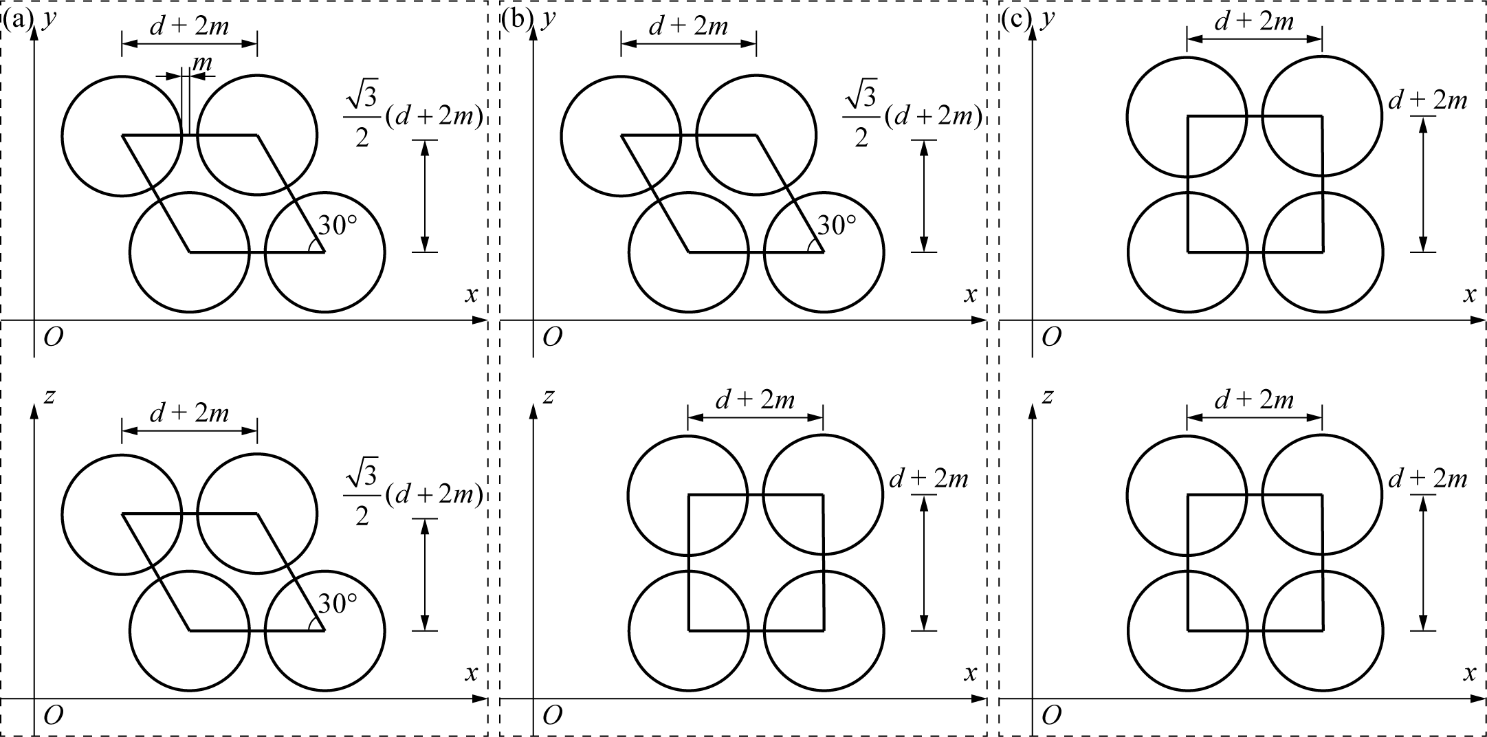


**Fig. 12.** Different particle arrangement sketch

According to the mass conservation, the relationship between unit weight of soil *γ′*_s_ and unit weight of particle *γ*_s_ can be obtained as follows.

$\frac{3}{4}\left(d+2m\right)^{3}\gamma'_{\text{s}}=\frac{\text{π}}{6}d^{3}\gamma_{\text{s}}$ (B1)

After simplification, constant *m* under the particle arrangement in Fig. 12(a) can be calculated by Eq. (B2).

$\frac{\gamma'_{\text{s}}}{\gamma_{\text{s}}}=\frac{2}{9}\text{π}\left(\frac{d}{d+2m}\right)^{3}$ (B2)

Similarly, *m* in Fig. 12(b) and (c) can be calculated by Eq. (B3) and (B4), respectively.

$\frac{\gamma'_{\text{s}}}{\gamma_{\text{s}}}=\frac{\sqrt{3}}{9}\text{π}\left(\frac{d}{d+2m}\right)^{3}$ (B3)

$\frac{\gamma'_{\text{s}}}{\gamma_{\text{s}}}=\frac{1}{6}\text{π}\left(\frac{d}{d+2m}\right)^{3}$ (B4)

The difference between the above three equations is the coefficient on the right side. This coefficient is related to the particle arrangement and the coefficient is concluded as $\frac{2}{9}\text{π(}1-\frac{m}{4\delta_{1}})$. Therefore, constant *m* could be obtained by Eq. (B5).

$\frac{\gamma'_{\text{s}}}{\gamma_{\text{s}}}=\frac{2}{9}\text{π}\left(1-\frac{m}{4\delta_{1}}\right)\left(\frac{d}{d+2m}\right)^{3}$ (B5)
